# A rare case of nephrotic syndrome revealing mycosis fungoide managed successfully with chemotherapy

**Published:** 2012-07-09

**Authors:** Mouna Kairouani, Sakina Sekkate, Nabil Ismaili, Halima Abahssain, Hassan Errihani

**Affiliations:** 1 Department of medical oncology, National Institute of oncology, Rabat, Morocco

**Keywords:** Nephrotic syndrome, mycosis fungoide, chemotherapy, multidisciplinary

## Abstract

The occurrence of the nephrotic syndrome during mycosis fungoide is very unusual. We report a rare case of mycosis fungoide revealed by hydrops related to nephrotic syndrom in a 37-year old male patient. He has been admitted to intensive care unit because of a breathing distress and a hydrophobs. Whole body computed tomography scan revealed bilateral axillary, cervical lymph nodes, tumoral infiltration of the subcutaneous tissue in the cervicothoracic and abdominal regions, multiples bilateral pulmonary metastasis, bilateral pleural effusion, and abdominal effusion; the kidneys were normal. The patient was staged IVb (T3N3M1). He was treated with CHOP (cyclophosphamide, Doxorubicin, Vincristin and prednisone). Evolution after eight cycles of chemotherapy was spectacular. The development of nephrotic syndrom secondary to mycosis fungoide is rare. It requires a multidisciplinary approach with nephrologists and oncologists.

## Introduction

Cutaneous T-cell lymphoma is a heterogenous group of non Hodgkin lymphoma including mycosis fungoide and sesary sundrome [1]. Mycosis fungoides is the most common disease entity which is characterized clinically by the presentation in the skin. Extracutaneous involvement is associated with poor prognosis, with a median survival less than 1.5 year [2]. The most commonly involved organs are lung, spleen, liver and gastrointestinal tract but Kidney involvement is rare in mycosis fungoide. It may be secondary to tumor involvement by the malignant proliferation or to immunologic mechanism [3]. We report a case of nephrotic syndrom revealing a mycosis fungoide.

## Patient and case report

A 37-year old man was admitted to intensive care unit because of a breathing distress and hydrophobs. He was found to have new onset hypertension and abnormal urianalysis. On physical examination, temperature was normal, pulse was 90 beat per minute and blood pressure was 190/110 mmHg. Erythematous follicular papule and tumefactions were evident all over his body and there was edema of the upper and lower limbs with an effusion of all serous. Furthermore, we found lymphadenopathy in axillary areas. Laboratory tests revealed a white blood cell count at 8 x 10^3^ g/L, hemoglobin was 12 g/dl, and platelet count 100x 10^3^/L. The serum creatinine was 4mg/l and urea level was 0,55g/l. Serum electrolytes were normal. A typical nephrotic syndrome was found: Urinary protein: 5g/day; serum total protein: 4.2g/day; and albumin: 27g/l. Urin electrophoresis, ANA, complement levels, and viral serologic findings were normal. Renal ultrasound showed normal kidneys. The renal biopsy was not done but biopsy specimens from the continuous lesions in the skin showed on morphology, small cells with similar nuclei, which infiltrated the epidermis, accompanied by interdigitating and Langerhan's cells. The cells of Mycosis fungoide expressed T-cell associated antigens CD3+, CD8+ and CD4+. The whole-body computed tomography scan revealed bilateral axillary, and cervical lymph nodes, tumoral infiltration of the subcutaneous tissue in the cervicothoracic and abdominal regions, multiples bilateral pulmonary metastasis, bilateral pleural effusion, and abdominal effusion. The patient was staged IVb (T3N3M1). He was immediately treated with CHOP (cyclophosphamide: 750mg/m^2^ in day 1; doxorubicin 50mg/m^2^ in day 1, vincristin 1.4mg/ m^2^ in day 1, and prednisone 40mg/ m^2^ in day 2 to 6) chemotherapy. He received 8 cycles of chemotherapy. Evolution was marked by a dramatic spectacular improvement of the symptoms, and the disappearance of the skin lesions. The blood pressure was normalized. Serum creatinine which initially peaked at 4 mg/dl, decrease dramatically to 1 mg/dl. Urine protein excretion decreased to < 300mg/24h. Serum albumin increased to 4g/dl. Repeated computed tomography of the abdomen after four and eight cycles of chemotherapy showed complete response. After a follow-up of 1 year, the patient has been admitted to intensive care unit because decompensated congestive heart failure and hydrophobs; he died before any medical workout.

## Discussion

Mycosis fungoides is the most common of the primary cutaneous lymphomas with an incidence rate of 4.1/1.000.000 person-years and male predominance. It constitutes 50% of all primary NHL of the skin [4]. The prognosis of MF is variable and strongly conditioned by the extent and type of skin involvement and the presence of extracutaneous disease. Visceral dissemination may develop subsequently after lymph nodes involvement, and the most commonly involved organs are the lungs, spleen, liver and gastrointestinal tract [5]. Renal involvement by the disease has rarely been reported. However, on autopsy, 16 to 31% of patients with mycosis fungoides have renal dissemination, most commonly with circumscribed malignant T–cell tumors or diffuse nodular infiltrates [6]. The current report describes a patient with nephrotic syndrome without individualization of renal tumor syndrom. Unfortunately, we couldn't perform a renal biopsy because of significant infiltration of the skin tissue but the paraneoplastic nature of glomerular disease is evident by the simultaneous evolution of lymphoma and renal involvement.

Paraneoplastic renal lesions are mainly glomerular. Predominant glomerular lesions may be suspected in the presence of proteinuria (especially over 1g/day) or nephrotic syndrome, microscopic or painless gross hematuria without blood clots, arterial hypertension, and/or decreased glomerular filtration rate and progressive renal disease. A nephrotic syndrome is defined by biological criteria: proteinuria greater than3g/day, hypoprotidemia (less than 60g/l) and hypoalbuminemia (less than 25 g/l), leading to edema. Percutaneous renal biopsy is mandatory to formally establish the diagnosis of the glomerulopathy, but it can be considered, depending of life expectancy [7]. Glomerular lesions revealed by nephrotic syndrom have been known to be associated with both chronic lymphocytic leukemia, and non hodgkin's lymphoma, particulary B cell lymphoma, but rarely described in T cell lymphoma. The pathogenesis of renal disease in lymphoma is poorly understood [8].

Shaloub first hypothesized that the T cell dysfunction is the consequence of a chemical mediator that caused glomerular dysfunction secondary to the alteration of the glomerular basement membrane [9]. Lagrue et al, cultured peripheral lymphocytes from patients having nephrotic syndrome and found an inflammatory lymphokine factor that enhanced vascular permeability [10].

The primary treatment of patients with cancer and paraneoplastic nephrotic syndrome should be directed at the cancer. The standard options in stage IV on mycosis fungoide is palliative chemotherapy. The most effective and commonly used combinations are CHOP (cyclophosphamide, Doxorubicin, Vincristin and prednisone), and CVP (Cyclophosphamide-vindésine-Prednisone) regimens. In spite of complete response rate of 80–100%, the median duration of response to chemotherapy is shorter than 1 year. The symptomatic treatment of the nephrotic syndrome by diuretics is justified. In the majority of patients, the use of furosemide with sodium and water restriction is sufficient. The association of nephrotic syndrome with mycosis fungoide seems to be a factor of poor prognosis. This justifies the adoption of multidisciplinary approach with nephrologists and oncologists.

## Conclusion

The development of nephrotic syndrom secondary to mycosis fungoides is rare. It should suggest a paraneoplastic syndrome that caused glomerular disease. The treatment is based on the treatment of the malignant lymphoma by chemotherapy.
